# Supplementary material for: The social organization of the Asian weaver ant colonies: A natural enemy novel sub-castes worker’s functional activity findings
Source: PLoS One. 2025 Jun 20;20(6):e0326030. doi: 10.1371/journal.pone.0326030 (PMC12180660; doi:10.1371/journal.pone.0326030)
Supplement: S1 Table — (DOCX) [file pone.0326030.s001.docx]

**S1 Table. Colony eggs production**

| ***Oecophylla* eggs** | | | | |
| --- | --- | --- | --- | --- |
| Colony | Nest No. 1 | Nest No. 2 | Total | Mean ± SD |
| 1 | 419 | 437 | 856 | 428.0 ± 12.7 |
| 2 | 423 | 431 | 834 | 417.0 ± 5.6 |
| 3 | 481 | 451 | 932 | 466.0 ± 21.2 |
| 4 | 413 | 421 | 834 | 417.0 ± 5.6 |
| 5 | 457 | 441 | 898 | 449.0 ± 11.3 |
| 6 | 450 | 445 | 895 | 447.5 ± 3.5 |
| 7 | 517 | 509 | 1026 | 513.0 ± 5.6 |
| 8 | 405 | 417 | 822 | 411.0 ± 8.4 |
| 9 | 479 | 491 | 970 | 485.0 ± 8.4 |
| 10 | 497 | 520 | 1017 | 508.5 ± 16.2 |
| Total | 4541 | 4563 | 9104 | 455.2 ± 15.5 |
