# Supplementary material for: The social organization of the Asian weaver ant colonies: A natural enemy novel sub-castes worker’s functional activity findings
Source: PLoS One. 2025 Jun 20;20(6):e0326030. doi: 10.1371/journal.pone.0326030 (PMC12180660; doi:10.1371/journal.pone.0326030)
Supplement: S2 Table — (DOCX) [file pone.0326030.s002.docx]

**S2 Table. Shapiro-Wilk normality test – Tukey HSD**

| Group | p-value | Sig./ Nsig. |  | Group | p-value | Sig./ Nsig. |  | Group | p-value | Sig./ Nsig. |
| --- | --- | --- | --- | --- | --- | --- | --- | --- | --- | --- |
| 2-1 | 1.0000 | Nsig. |  | **9-2** | **0.0091** | **Sig.** |  | 6-5 | 1.0000 | Nsig. |
| 3-1 | 0.1128 | Nsig. |  | **10-2** | **0.0007** | **Sig.** |  | **7-5** | **0.0044** | **Sig.** |
| 4-1 | 0.9870 | Nsig. |  | **4-3** | **0.0277** | **Sig.** |  | 8-5 | 0.1128 | Nsig. |
| 5-1 | 0.6854 | Nsig. |  | 5-3 | 0.8589 | Nsig. |  | 9-5 | 0.1447 | Nsig. |
| 6-1 | 0.7557 | Nsig. |  | 6-3 | 0.7996 | Nsig. |  | **10-5** | **0.0076** | **Sig.** |
| **7-1** | **0.0005** | **Sig.** |  | **7-3** | **0.0358** | **Sig.** |  | **7-6** | **0.0037** | **Sig.** |
| 8-1 | 0.8589 | Nsig. |  | **8-3** | **0.0131** | **Sig.** |  | 8-6 | 0.1360 | Nsig. |
| **9-1** | **0.0102** | **Sig.** |  | 9-3 | 0.7780 | Nsig. |  | 9-6 | 0.1201 | Nsig. |
| **10-1** | **0.0007** | **Sig.** |  | 10-3 | 0.0636 | Nsig. |  | **10-6** | **0.0063** | **Sig.** |
| 3-2 | 0.0994 | Nsig. |  | 5-4 | 0.2346 | Nsig. |  | **8-7** | **0.0001** | **Sig.** |
| 4-2 | 0.9931 | Nsig. |  | 6-4 | 0.2788 | Nsig. |  | 9-7 | 0.3666 | Nsig. |
| 5-2 | 0.6370 | Nsig. |  | **7-4** | **0.0002** | **Sig.** |  | 10-7 | 1.0000 | Nsig. |
| 6-2 | 0.7092 | Nsig. |  | 8-4 | 0.9999 | Nsig. |  | **9-8** | **0.0014** | **Sig.** |
| **7-2** | **0.0004** | **Sig.** |  | **9-4** | **0.0028** | **Sig.** |  | **10-8** | **0.0001** | **Sig.** |
| 8-2 | 0.8929 | Nsig. |  | **10-4** | **0.0002** | **Sig.** |  | 10-9 | 0.5644 | Nsig. |

| Shapiro-Wilk normality test  data: dt$Nest  W = 0.92215, p-value = 0.109  ## ANOVA  model_anova <- aov(Nest ~ as.factor(Colony), data=dt)  summary(model_anova)  > summary(model_anova)  Df Sum Sq Mean Sq F value Pr(>F)  as.factor(Colony) 9 24464 2718.2 21.62 2.06e-05 ***  Residuals 10 1257 125.7  ---  Signif. codes: 0 ‘***’ 0.001 ‘**’ 0.01 ‘*’ 0.05 ‘.’ 0.1 ‘ ’ 1 |
| --- |
| # Multiple Comparisons  TukeyHSD(model_anova, conf.level = 0.95)  Tukey multiple comparisons of means  95% family-wise confidence level |
| ## Normality test for Residual  shapiro.test(resid(model_anova))  > shapiro.test(resid(model_anova))  Shapiro-Wilk normality test  data: resid(model_anova)  W = 0.97195, p-value = 0.7954 |
