# Supplementary material for: The social organization of the Asian weaver ant colonies: A natural enemy novel sub-castes worker’s functional activity findings
Source: PLoS One. 2025 Jun 20;20(6):e0326030. doi: 10.1371/journal.pone.0326030 (PMC12180660; doi:10.1371/journal.pone.0326030)
Supplement: S3 Table — (DOCX) [file pone.0326030.s003.docx]

**S3 Table. Box-Cox Transformation**

Since the data is not normally distributed, the data transformation might be needed. Hence, the box-cox method is applied to obtain the lambda value to determine the suitable data transformation.

| library(MASS)  x <- dt$HW  x <- dt$HL  x <- dt$TL  x <- dt$AL  x <- dt$BL  # Fit a simple intercept-only model  model <- lm(x ~ 1)  # Apply Box-Cox transformation and get lambda values  bc <- boxcox(model, lambda = seq(-2, 2, 0.1))  # Identify the lambda that maximizes log-likelihood  max_lambda <- bc$x[which.max(bc$y)]  max_lambda |
| --- |
| HW: lambda= 1.313131 close to 1, no transformation needed  HL: lambda=1.717172 close to 2, transform x^2  TL: lambda=1.353535 close to 1, no transformation needed  AL: lambda=0.2626263 close to 0, transform log(x)  BL: lambda=0.7474747 close to 1, no transformation needed |

| # Transformed data  new_HL <- x^2  # Histogram  hist(dt$HL)  shapiro.test(dt$HL)  hist(new_HL)  shapiro.test(new_HL) | |
| --- | --- |
| 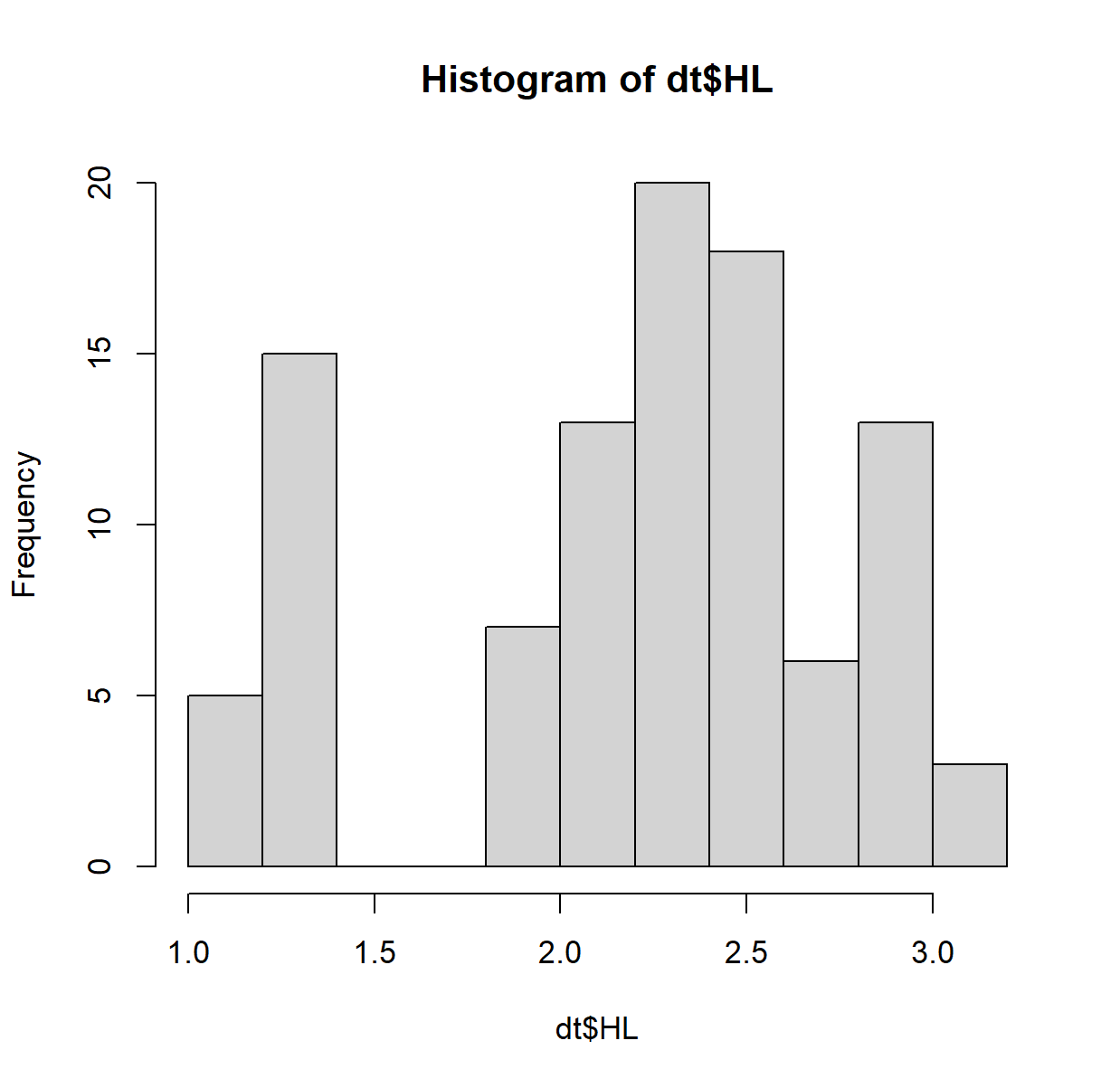  > shapiro.test(dt$HL)  Shapiro-Wilk normality test  data: dt$HL  W = 0.92493, p-value = 2.597e-05 | 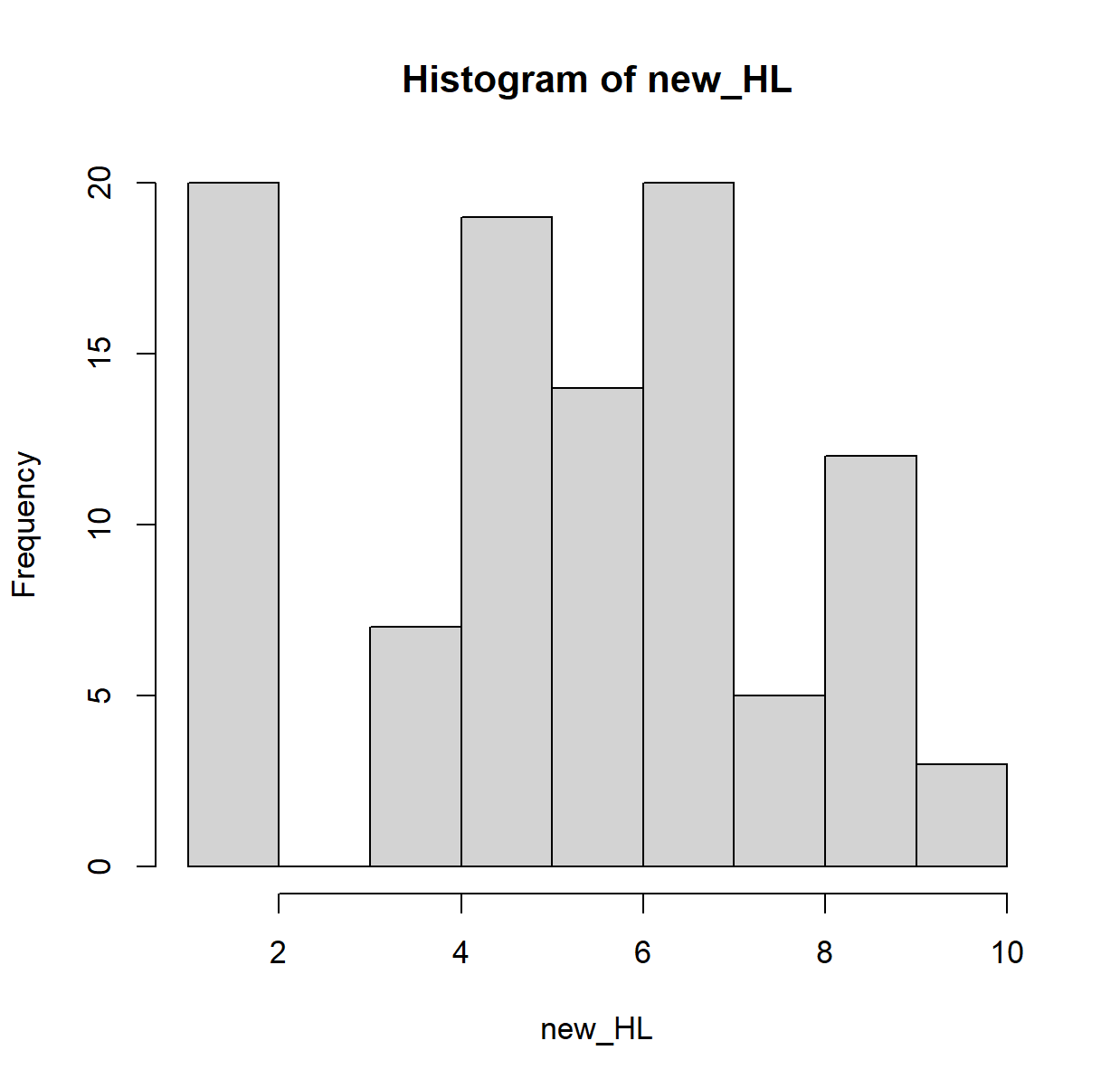  > shapiro.test(new_HL)  Shapiro-Wilk normality test  data: new_HL  W = 0.95074, p-value = 0.0009291  Comment: The effect of transformation is not significant. |
| # Transformed data  new_AL <- log(x)  # Histogram  hist(dt$AL)  shapiro.test(dt$AL)  hist(new_AL)  shapiro.test(new_AL) | |
| 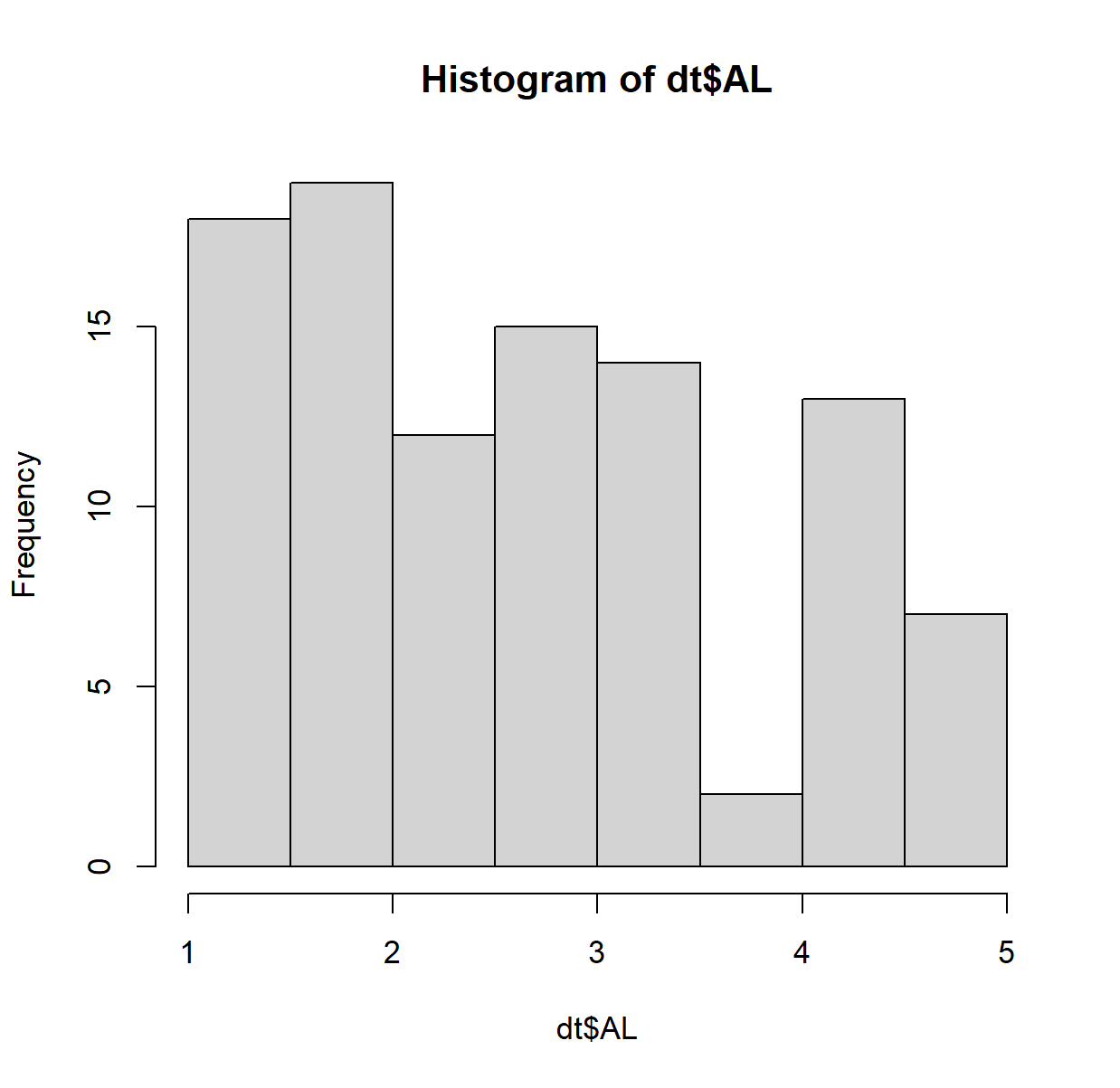  > shapiro.test(dt$AL)  Shapiro-Wilk normality test  data: dt$AL  W = 0.94456, p-value = 0.0003697 | **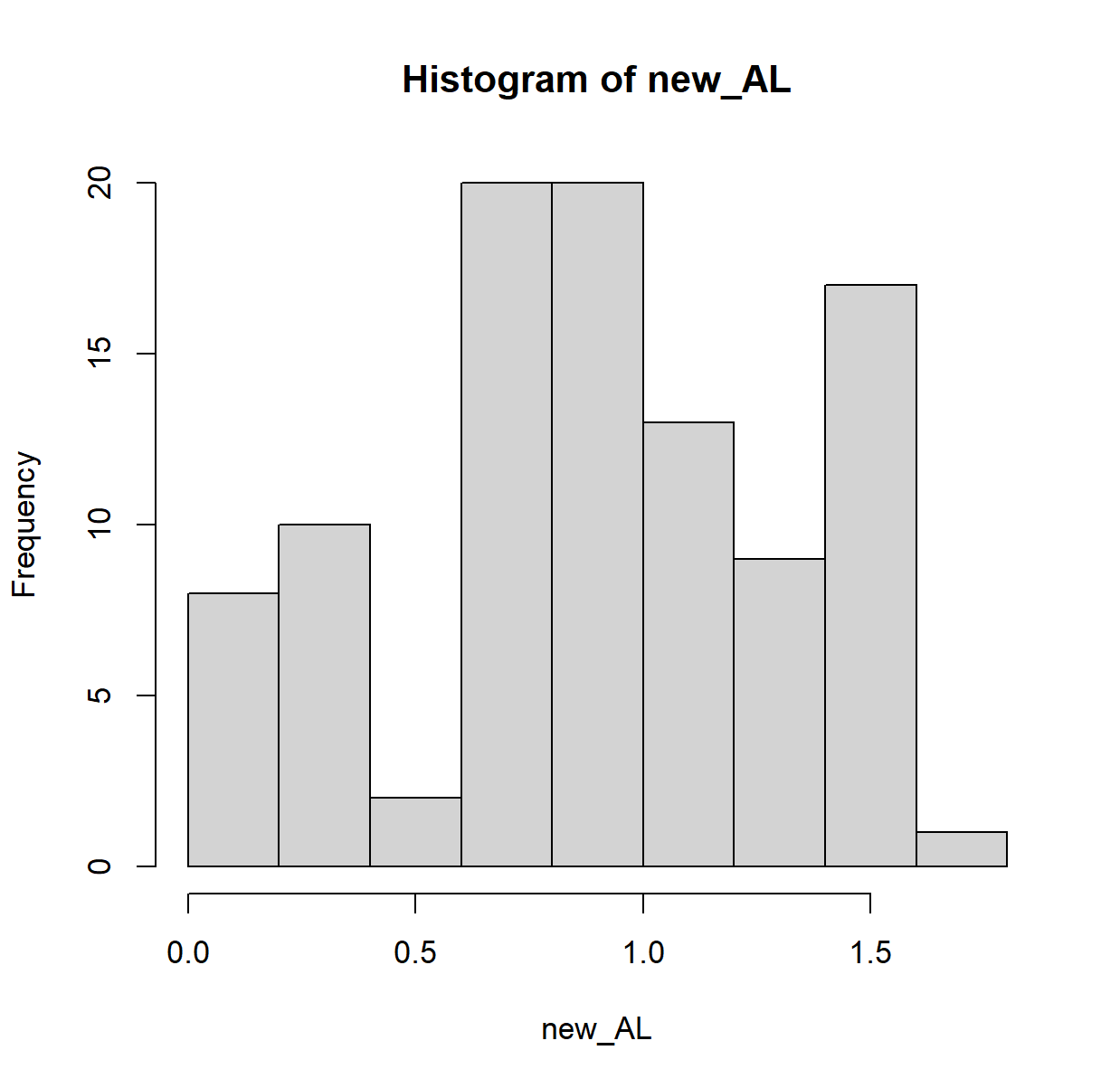**  **>** shapiro.test(new_AL)  Shapiro-Wilk normality test  data: new_AL  W = 0.95543, p-value = 0.001924  Comment: The effect of transformation is not significant. |
