# Supplementary material for: The social organization of the Asian weaver ant colonies: A natural enemy novel sub-castes worker’s functional activity findings
Source: PLoS One. 2025 Jun 20;20(6):e0326030. doi: 10.1371/journal.pone.0326030 (PMC12180660; doi:10.1371/journal.pone.0326030)
Supplement: S4 Table — Sub-castes. (DOCX) [file pone.0326030.s004.docx]

**S4 Table. Descriptive statistics. Sub-castes**

| Ant Worker Group | Dimension (in mm) |
| --- | --- |
| MBM = Major Big Worker  MIM = Major Intermediate Worker  IW3 = Intermediate Worker III  IW2 = Intermediate Worker II  MW = Minor Worker | Head width (HW)  Head length (HL)  Thorax length (TL)  Abdomen length (AL)  Body length (BL) |

**Data Structure**

| > head(dt)  HW HL TL AL BL Type  1 2.12 3.15 3.90 5.00 12.8 MBW  2 2.10 3.15 3.90 4.90 12.7 MBW  3 2.07 3.10 3.90 4.90 12.6 MBW  4 2.05 3.00 3.85 4.85 12.5 MBW  5 2.05 3.00 3.77 4.70 12.3 MBW  6 2.02 3.00 3.75 4.65 12.2 MBW | > tail(dt)  HW HL TL AL BL Type  95 0.65 1.30 1.36 1.23 4.54 MW  96 0.73 1.32 1.43 1.40 4.88 MW  97 0.71 1.06 1.34 1.30 4.41 MW  98 0.69 1.24 1.37 1.42 4.72 MW  99 0.72 1.25 1.29 1.10 4.36 MW  100 0.71 1.27 1.28 1.16 4.34 MW |
| --- | --- |
| > dt[, 'Type'] <- as.factor(dt[, 'Type'])  > str(dt)  'data.frame': 100 obs. of 6 variables:  $ HW : num 2.12 2.1 2.07 2.05 2.05 2.02 2.03 2.05 2.07 2.01 ...  $ HL : num 3.15 3.15 3.1 3 3 3 3 3 3 2.9 ...  $ TL : num 3.9 3.9 3.9 3.85 3.77 3.75 3.6 3.5 3.48 3.44 ...  $ AL : num 5 4.9 4.9 4.85 4.7 4.65 4.6 4.35 4.33 4.2 ...  $ BL : num 12.8 12.7 12.6 12.5 12.3 ...  $ Type: Factor w/ 5 levels "IW2","IW3","MBW",..: 3 3 3 3 3 3 3 3 3 3 ... | |
| > levels(dt$Type)  [1] "IW2" "IW3" "MBW" "MIW" "MW" | |

**Descriptive Statistics**

| **Descriptive Statistics by Ant’s Dimensions** |
| --- |
| > describe(dt[,-6])  vars n mean sd median trimmed mad min max range skew kurtosis se  HW 1 100 1.41 0.46 1.52 1.42 0.47 0.61 2.12 1.51 -0.34 -1.15 0.05  HL 2 100 2.21 0.57 2.26 2.23 0.44 1.06 3.15 2.09 -0.48 -0.70 0.06  TL 3 100 2.62 0.76 2.78 2.64 0.73 1.28 3.90 2.62 -0.31 -0.91 0.08  AL 4 100 2.66 1.09 2.51 2.60 1.03 1.10 5.00 3.90 0.42 -0.86 0.11  BL 5 100 8.14 2.65 7.97 8.10 3.10 3.91 12.75 8.84 0.01 -1.22 0.26  **Note:** The skewness values are **-0.5<skewness<0.5,** so it is **approximately symmetry (normal).** |
| **Descriptive Statistics by Ant’s Worker Types** |
| > describeBy(dt$HW, dt$Type) # Head width  Descriptive statistics by group  group: IW2  vars n mean sd median trimmed mad min max range skew kurtosis se  X1 1 20 1.16 0.19 1.23 1.17 0.2 0.82 1.4 0.58 -0.45 -1.39 0.04  ----------------------------------------------------------  group: IW3  vars n mean sd median trimmed mad min max range skew kurtosis se  X1 1 20 1.52 0.08 1.54 1.52 0.04 1.32 1.7 0.38 -0.28 0.81 0.02  ----------------------------------------------------------  group: MBW  vars n mean sd median trimmed mad min max range skew kurtosis se  X1 1 20 1.99 0.07 2.01 1.99 0.09 1.89 2.12 0.23 -0.02 -1.52 0.02  ----------------------------------------------------------  group: MIW  vars n mean sd median trimmed mad min max range skew kurtosis se  X1 1 20 1.68 0.11 1.67 1.68 0.13 1.46 1.87 0.41 -0.04 -1.04 0.02  ----------------------------------------------------------  group: MW  vars n mean sd median trimmed mad min max range skew kurtosis se  X1 1 20 0.7 0.03 0.71 0.7 0.03 0.61 0.75 0.14 -1.02 0.47 0.01 |
