# Supplementary material for: The social organization of the Asian weaver ant colonies: A natural enemy novel sub-castes worker’s functional activity findings
Source: PLoS One. 2025 Jun 20;20(6):e0326030. doi: 10.1371/journal.pone.0326030 (PMC12180660; doi:10.1371/journal.pone.0326030)
Supplement: S5 Table — (DOCX) [file pone.0326030.s005.docx]

**S5 Table. Correlation Comparison by Ant Worker Sub-Caste - Spearman Correlation versus Pearson Correlation**

| **Spearman Correlation** | **Pearson Correlation** |
| --- | --- |
| ### Spearman Correlation Analysis for MBW ###  Correlation Coefficients:  HW HL TL AL BL  HW 1.0000 0.9647 0.8817 0.9434 0.9403  HL 0.9647 1.0000 0.9325 0.9729 0.9752  TL 0.8817 0.9325 1.0000 0.9389 0.9453  AL 0.9434 0.9729 0.9389 1.0000 0.9966  BL 0.9403 0.9752 0.9453 0.9966 1.0000 | ### Pearson Correlation Analysis for MBW ###  Correlation Coefficients:  HW HL TL AL BL  HW 1.0000 0.9661 0.8664 0.8730 0.9427  HL 0.9661 1.0000 0.9134 0.9249 0.9722  TL 0.8664 0.9134 1.0000 0.9093 0.9431  AL 0.8730 0.9249 0.9093 1.0000 0.9695  BL 0.9427 0.9722 0.9431 0.9695 1.0000 |
| ### Spearman Correlation Analysis for MIW ###  Correlation Coefficients:  HW HL TL AL BL  HW 1.0000 0.6526 0.0369 0.1084 0.2636  HL 0.6526 1.0000 0.1189 0.1855 0.4018  TL 0.0369 0.1189 1.0000 0.6154 0.3031  AL 0.1084 0.1855 0.6154 1.0000 0.2960  BL 0.2636 0.4018 0.3031 0.2960 1.0000 | ### Pearson Correlation Analysis for MIW ###  Correlation Coefficients:  HW HL TL AL BL  HW 1.0000 0.5223 0.0233 0.1211 0.3224  HL 0.5223 1.0000 -0.1122 0.0296 0.2221  TL 0.0233 -0.1122 1.0000 0.5546 0.1550  AL 0.1211 0.0296 0.5546 1.0000 0.3230  BL 0.3224 0.2221 0.1550 0.3230 1.0000 |
| ### Spearman Correlation Analysis for IW3 ###  Correlation Coefficients:  HW HL TL AL BL  HW 1.0000 0.2026 0.3374 0.1641 0.6444  HL 0.2026 1.0000 0.4077 0.1293 0.5163  TL 0.3374 0.4077 1.0000 0.1444 0.2208  AL 0.1641 0.1293 0.1444 1.0000 0.0340  BL 0.6444 0.5163 0.2208 0.0340 1.0000 | ### Pearson Correlation Analysis for IW3 ###  Correlation Coefficients:  HW HL TL AL BL  HW 1.0000 -0.0740 0.2544 0.0122 0.6019  HL -0.0740 1.0000 0.3449 0.1027 0.3615  TL 0.2544 0.3449 1.0000 0.2217 0.2509  AL 0.0122 0.1027 0.2217 1.0000 0.0636  BL 0.6019 0.3615 0.2509 0.0636 1.0000 |
| ### Spearman Correlation Analysis for IW2 ###  Correlation Coefficients:  HW HL TL AL BL  HW 1.0000 0.0727 -0.1125 0.0053 0.4329  HL 0.0727 1.0000 -0.1530 0.0903 -0.0373  TL -0.1125 -0.1530 1.0000 0.1397 0.1394  AL 0.0053 0.0903 0.1397 1.0000 0.6592  BL 0.4329 -0.0373 0.1394 0.6592 1.0000 | ### Pearson Correlation Analysis for IW2 ###  Correlation Coefficients:  HW HL TL AL BL  HW 1.0000 0.0540 -0.1475 0.1168 0.4387  HL 0.0540 1.0000 -0.1300 0.1411 -0.1137  TL -0.1475 -0.1300 1.0000 0.0511 0.1144  AL 0.1168 0.1411 0.0511 1.0000 0.7117  BL 0.4387 -0.1137 0.1144 0.7117 1.0000 |
| ### Spearman Correlation Analysis for MW ###  Correlation Coefficients:  HW HL TL AL BL  HW 1.0000 0.2854 0.2593 0.0519 0.3320  HL 0.2854 1.0000 0.4621 0.4795 0.1969  TL 0.2593 0.4621 1.0000 0.6261 -0.0964  AL 0.0519 0.4795 0.6261 1.0000 0.1548  BL 0.3320 0.1969 -0.0964 0.1548 1.0000 | ### Pearson Correlation Analysis for MW ###  Correlation Coefficients:  HW HL TL AL BL  HW 1.0000 0.1144 0.2641 0.1758 0.3235  HL 0.1144 1.0000 0.3396 0.3858 0.1782  TL 0.2641 0.3396 1.0000 0.5293 -0.1905  AL 0.1758 0.3858 0.5293 1.0000 0.0112  BL 0.3235 0.1782 -0.1905 0.0112 1.0000 |
