# Supplementary material for: The social organization of the Asian weaver ant colonies: A natural enemy novel sub-castes worker’s functional activity findings
Source: PLoS One. 2025 Jun 20;20(6):e0326030. doi: 10.1371/journal.pone.0326030 (PMC12180660; doi:10.1371/journal.pone.0326030)
Supplement: S6 Table — (DOCX) [file pone.0326030.s006.docx]

**S6 Table. Test of Multicollinearity**

| **Predictors** | **Significant Predictors**  p-value < 0.05 | **Normality Test**  **for residual**  **Shapiro-Wilks**  p-value > 0.05 | **VIF^a^**  VIF < 5, no multicol  5 < VIF < 10, moderate multicol  VIF > 10, high multicol  VIF > 20, severe multicol |
| --- | --- | --- | --- |
| HW | HW  R2 = 0.93 | p-value = 0.3 > 0.05 | NA |
| HL | HL  R2 = 0.86 | p-value = 1e-04  < 0.05 | NA |
| TL | TL  R2 = 0.93 | p-value = 0.02  < 0.05 | NA |
| AL | AL  R2 = 0.96 | p-value = 0.003  < 0.05 | NA |
| HW, HL | HW, HL  R2 = 0.94 | p-value = 0.02  < 0.05 | HW HL  7.53 7.53 |
| HW, TL | HW, TL  R2 = 0.95 | p-value = 0.004  < 0.05 | HW TL  10.1 10.1 |
| HW, AL | HW, AL  R2 = 0.98 | p-value = 0.02  < 0.05 | HW AL  7.66 7.66 |
| HL, TL | HL, TL  R2 = 0.93 | p-value = 8e-05  < 0.05 | HL TL  8.63 8.63 |
| HL, AL | HL, AL  R2 = 0.97 | p-value = 0.003  < 0.05 | HL AL  5.66 5.66 |
| TL, AL | TL, AL  R2 = 0.97 | p-value = 0.8  > 0.05 | TL AL  8.65 8.65 |
| HW, HL, TL | HW, TL  R2 = 0.95 | p-value = 0.003  < 0.05 | HW HL TL  11.60 9.93 13.29 |
| HW, HL, AL | HW, AL  R2 = 0.98 | p-value = 0.01  < 0.05 | HW HL AL  11.17 8.26 8.40 |
| HW, TL, AL | HW, TL, AL  R2 = 0.98 | p-value = 0.2  > 0.05 | HW TL AL  11.7 13.2 10.0 |
| HL, TL, AL | TL, AL  R2 = 0.98 | p-value = 0.4 > 0.05 | HL TL AL  8.98 13.74 9.01 |
| HW, HL, TL, AL | HW, AL, TL  R2 = 0.98 | p-value = 0.2 > 0.05 | HW HL TL AL  13.00 9.99 15.99 10.10  HL has moderate multicol. |

**^a^**O’Brien (2007) demonstrated that high VIF showing high multicollinearity does not necessarily invalidate, nor contradict the regression findings.
