# Supplementary material for: The social organization of the Asian weaver ant colonies: A natural enemy novel sub-castes worker’s functional activity findings
Source: PLoS One. 2025 Jun 20;20(6):e0326030. doi: 10.1371/journal.pone.0326030 (PMC12180660; doi:10.1371/journal.pone.0326030)
Supplement: S7 Table — (DOCX) [file pone.0326030.s007.docx]

**S7 Table**. **Descriptive analysis of *O. smaragdina* Felda I colony**

| **Variables** | **Nest one** | **Nest two** | **Nest three** | **Total nest** | | |
| --- | --- | --- | --- | --- | --- | --- |
|  |  |  |  | **Min** | **Max** | **Mean ±SD** |
| Number of leaflets | 8 | 8 | 9 | 8 | 9 | 8.33 ± 0.5 |
| Nest length | 57.0 | 47.0 | 60.0 | 47.0 | 60.0 | 54.6 ± 11.5^a^ |
| Nest width | 25.0 | 11.0 | 27.0 | 11.0 | 27.0 | 21.0 ± 7.0^a^ |
| Nest height | 15.0 | 9.0 | 20.0 | 2.0 | 23.0 | 14.6 ± 8.5^a^ |
| ***Height from the ground | 6.0 | 8.0 | 8.0 | 6.0 | 8.0 | 7.33 ± 1.15 |
| Total workers | 14279 | 4985 | 16623 | 4985 | 16623 | 11962 ±6155 |
| Number of major workers  **Number of intermediate workers**** | 5753  **4175** | 2009  **1458** | 6379  **5101** | 2009  **1458** | 5753  **5101** | 4713 ±2363  **3578 ±1893** |
| Number of minor workers | 4351 | 3721 | 5143 | 3721 | 5143 | 1387 **±** 1098^a^ |
| Number of winged green queens  Number of newly emerged queens* | 17  5 | 15  9 | 19  11 | 15  5 | 19  11 | 17 ± 2  8.33 ±3 |
| Number of drone males : 0  Number of worker pupae | 1255 | 342 | 357 | 15 | 2038 | 878 ± 792^a^ |
| Number of larvae | 854.0 | 427.0 | 1375.0 | 427.0 | 1375.0 | 885.3 ±474.7 |
| Egg volume | 3.1 | 4.5 | 15.2 | 3.1 | 15.2 | - 1. ± 1.6^a^ |

Queen larvae 21.0 15.0 0.0 0 .0 21.0 12 ±10.8

Male larvae 37.0 29.0 0.0 0.0 37.0 22 ±19.4

Eggs count 1874 4890 0 0 4890 2254 ±2515

Note: ^a^Variables with a different superscript alphabet had significantly different mean values at p < 0.05

*Callow yellow queens becoming green after 7 days - **novel workers caste - ***Barrack nest range 2-4 m height to the ground.
