# Supplementary material for: The social organization of the Asian weaver ant colonies: A natural enemy novel sub-castes worker’s functional activity findings
Source: PLoS One. 2025 Jun 20;20(6):e0326030. doi: 10.1371/journal.pone.0326030 (PMC12180660; doi:10.1371/journal.pone.0326030)
Supplement: S8 Table — (DOCX) [file pone.0326030.s008.docx]

**S8 Table. Descriptive analysis of *O. smaragdina* matured big size colony UM III**

| **Variables** | **Colony (3 nests)** | | | |
| --- | --- | --- | --- | --- |
|  | **Min** | **Max** | **Mean ±SD** | |
| Number of leaflets | 6 | 19 | | 13.33 ± 4^a^ |
| Nest length | 30.0 | 60.0 | | 49.0 ± 12.3^a^ |
| Nest width | 8.0 | 30.0 | | 17.0 ± 7.0^a^ |
| Nest height | 2.0 | 23.0 | | 10.4 ± 6.7^a^ |
| Nest volume | 829.4 | 16618.5 | | 5889.1 ± 6347.9^a^ |
| Height from the ground | 9.0 | 10.0 | | 9.6 ± 0.5^a^ |
| Total workers | 2448 | 64761 | | 16432 ± 20896^a^ |
| Number of major workers  Number of intermediate workers | 1205  201 | 40873  19102 | | 15045 ± 19828^a^  6723.3 ±20170 |
| Number of minor workers | 376 | 4786 | | 1387 ± 1098^a^ |
| Number of winged queens*  Number of newly emerged queens**  Number of males^1^ | 0.0  10.0  0 | 138.0  33.0  0 | | 64.6 ± 63.9^b^  21.3 ±11.5  0 |
| Number of worker pupae  Queen larvae  Male larvae | 154  0.0  0 | 2038  37.0  49 | | 918 ± 991^a^  22 ±19.4  29.6 ±26 |
| Number of workers larvae | 71.0 | 6509.0 | | 2821.6 ± 3319.6^a^ |
| Egg volume  Eggs count | 0.0  0 .0 | 4.7  3845.0 | | 1.0 ± 1.6^a^  1505.6 ± 2053.6 |

Note: *Green colour gradually reaching a week of development; ** Emergence as yellow pale colour changing gradually to green upon high water consumption; ^a^Variables with the different superscript alphabet had significantly different mean values at p < 0.05

^1^An average of 300 drone reproductive males were found in two big nests in Saratok, Sarawak Borneo MPOB.
