# Supplementary material for: The social organization of the Asian weaver ant colonies: A natural enemy novel sub-castes worker’s functional activity findings
Source: PLoS One. 2025 Jun 20;20(6):e0326030. doi: 10.1371/journal.pone.0326030 (PMC12180660; doi:10.1371/journal.pone.0326030)
Supplement: Attachment — Submitted filename: Response to Reviewers PONE-D-24-57771.docx [file pone.0326030.s010.docx]

**Response to Reviewers**

**PONE-D-24-57771**

**The social organization of the Asian weaver ant colonies: A natural enemy novel sub-castes worker’s functional activity findings**

**PLOS ONE**

On behalf of all authors, I would like to thank all the reviewers and academic editors of PlosOne for their valuable feedback. The review from the Academic Editor and the two anonymous reviewers have helped tremendously to improve the manuscript overall quality and content.

**Academic Editor – journal requirements**

**Based on both review report, there looks major concern about the duplication of findings with the pre-print as highlighted by the reviewer. Guidelines for improvement must be followed before submission of revised manuscript.
Statistical analysis needs major revision and must based on the parameters as suggested by the reviewers.
Figures unclear must be rechecked in the revised manuscript.
All suggestions point-wise must be answered properly.**

**Point 1. Manuscript formatting-editing following PlosOne requirements.**

The journal has been formatted accordingly.

**Point 2. Please include a copy of Table 4 which you refer to in your text on page 16.**

Table 4 mentioned in the text was a mistake and has been replaced by Figs 14 A-B in the discussion page 16.

**Point 3. ‘Please include captions for your Supporting Information files at the end of your manuscript, and update any in-text citations to match accordingly.**

Captions for supporting information are included at the end of the manuscript as per journal request.

**Point 4. Statistical analysis.**

Statistical analyses are done according to the reviewer's requirements. We proposed an added method with similarity to GLM called Generalized Estimating Equations (GEE) model. Revised the overall analyses because we forgot to standardize the final data set. Results are slightly different but better now.

**Point 5. Figures**

Figures have been rechecked and revised accordingly thank you.

All suggestions point-wise are addressed below.

Thank you.

**Reviewer 1.**

**Point 1. Abstract. Reviewer #1: In the abstract: summary should be provided, without writing anything not known by the readers e.g. significant correlation - first report ?? Rewrite the abstract, without any unnecessary information.**

The abstract has been revised accordingly, following the recommendations given by the reviewer 1. Thank you.

**Point 2. ‘Similarity with online preprint**

**I found the following publication on** [**researchgate.net**](http://researchgate.net/)**, which has almost similar content but posted to "preprints".
Please remove the same content from this manuscript and re-write this paper including the findings "NEW" ONLY here. Although the titles are different, many sections in both manuscripts overlap.**

**The social organisation of Oecophylla smaragdina (Hymenoptera:
Formicidae) colonies and their functional activity
Moïse Pierre Exélis1, 2, Azarae Hj Idris1, Zubaidah Ya’cob3, Rabha W. Ibrahim4, 5, 6, Salmah Yaakop7,
Nurul Wahida Othman7, Rosli Ramli1***

This preprint is the original work for this research and has never been published. It was understood that preprints do not have an impact on the issue of duplicates. It is the first research ever performed on this topic, and impossible to change the text content as it would not properly provide the right complete information to the readers. The least that can be done if the present manuscript is accepted is to delete this document from ResearchGate. And it is showing an ongoing study that protects us from any false authorship claims from others (study copy and duplicate). That is not an officially endorsed publication (Research Gate), in the present form, it was never peer-reviewed yet, but an original draft. I was obliged as a student to do the following to prevent the act of stealing a study idea and content. In the past, I had to face such a reality due to competition for the subject on the Asian weaver ants.

**Point 3. Pearson correlation and normal data distribution.**

**Statistical analysis:

Pearson's correlation should be applied to normally distributed data. your data normally distributed? Did you check?**

The Shapiro-Wilk test for normality is applied, and it is found that a majority of body dimensions are normally distributed, while others are not. For simplicity at this stage, we assume that the data is normally distributed. In this study, Pearson’s correlation is used. If not, the Spearman's rank correlation coefficient can be used to calculate the correlation.

Normality Test: Shapiro-Wilk method is used to test for data normality.

Shapiro Wilks Test: If p-value > 0.05, fail to reject H₀ → Data is normally distributed

| Ant Type | Dimension | p-value | Comment |
| --- | --- | --- | --- |
| MBW | Head width | 0.0995 | 1. 15 out of 25 ant worker dimensions are normally distributed. 2. 10 out of 25 ant worker dimensions are not normally distributed. 3. Since some variables are not normally distributed, ANOVA (parametric) is not preferred for comparing the means across different ant worker types. 4. Instead, the **Kruskal-Wallis test (non-parametric) is used to assess median equality**, as some variables are non-normally distributed, with p-values < 0.05. 5. Data homogeneity or equal variances must be checked for both normal and non-normal data using Levene's test. |
|  | Head length | 0.0178 |  |
|  | Thorax length | 0.0215 |  |
|  | Abdomen length | 0.0048 |  |
|  | Body length | 0.0663 |  |
| MIW | Head width | 0.7372 |  |
|  | Head length | 0.0924 |  |
|  | Thorax length | 0.8501 |  |
|  | Abdomen length | 0.2048 |  |
|  | Body length | 0.0814 |  |
| IW2 | Head width | 0.2376 |  |
|  | Head length | 0.0250 |  |
|  | Thorax length | 0.0142 |  |
|  | Abdomen length | 0.4445 |  |
|  | Body length | 0.2073 |  |
| IW3 | Head width | 0.0228 |  |
|  | Head length | 0.0073 |  |
|  | Thorax length | 0.6426 |  |
|  | Abdomen length | 0.0114 |  |
|  | Body length | 0.3680 |  |
| MW | Head width | 0.0448 |  |
|  | Head length | 0.2733 |  |
|  | Thorax length | 0.0866 |  |
|  | Abdomen length | 0.0371 |  |
|  | Body length | 0.5429 |  |

Furthermore, based on the descriptive statistics, it was found that the skewness is within -0.5 and 0.5. Hence, the data can be considered normally distributed.

| Variable | Mean | Median | Stdev | Skewness |
| --- | --- | --- | --- | --- |
| HW | 1.41 | 0.46 | 1.52 | -0.34 |
| HL | 2.21 | 0.57 | 2.26 | -0.48 |
| TL | 2.62 | 0.76 | 2.78 | -0.31 |
| AL | 2.66 | 1.09 | 2.51 | 0.42 |
| BL | 8.14 | 2.65 | 7.97 | 0.01 |

**Point 4. Kruskal Wallis plot.**

**Fig. 9: Kruskal Wallis mean plot - Is this a mean plot? Kruskal Wallis is about the median.**

You are right that Kruskal Wallis (non-parametric test) is a median plot. I made a mistake in labelling the figure.

**Point 5. Confirmation of novel sub-caste existence.**

**About intermediate worker castes mentioned in the manuscript:
Are you sure these are not "callow" major and minor workers? After the emergence from respective pupae, worker appearance including the size is different from that of mature workers (in most of the ant colonies including *O. smaragdina*, I observed this; also there are publications on other ant species.)**

First, this aspect can only be validated from mature colonies, but never from incipient colonies.

Yes, I am strongly sure that these are sub-caste intermediate worker classes. It was already reported, as mentioned in the manuscript (citations are provided). I have been observing the Asian weaver ant polymorphs workers since 2010, extensively in the field, with a couple of laboratory colony rearing.

Another report highlighted the extant third intermediate worker, whose average size is double that of minors and one-half that of majors (Vidhu & Evans, 2011). The head width, length, thorax, body, and abdomen length were not evaluated. Vanderplank (1960) did report this for the African weaver ant but without enough detailed data.

Vanderplank, F. L. (1960). The Bionomics and Ecology of the Red Tree Ant, *Oecophylla* sp., and its Relationship to the Coconut Bug *Pseudotheraptus wayi* Brown (Coreidae). *Journal of Animal Ecology*, *29*(1), 15-33.

<https://www.jstor.org/stable/2268>

The introduction section title “1.1 **Colony’s caste social structure composition”** explains this approach, citing Trible & Kronauer, 2017, and Kronauer et al, 2017.

Anand et al’s (2022) strong stand on the genetically heritable traits rather than the morphological variation in shape and size, enforced my point.

The individuals were closely monitored from the larval stage, pupae, up to their emergence as adults. Photos taken, measurements recorded. Visually, the body and the abdomen have a shape, size that is striking. After repeatedly sampling over the years, 1000s of individuals following the same features, it was clear to me that these are novel findings.

Generally, among most ant species, inter-caste or sub-caste of individuals intermediate in size between the castes is scarce or absent (Kronauer et al., 2017).

A recent report in Biological Control journal by Karthick et al., 2024 highlighted about polymorphs workers based on the antenna and leg apparatus. However, we are curious to see a set of photos of each individual identified as different from the others.

KARTHICK M, SAMUEL B, SELVAM IK, RANI CS, RAJ RA. Ultrastructure and morphometric characteristics of *Oecophylla smaragdina* (Fabricius, 1775) (Hymenoptera: Formicidae). Journal of Biological Control. 2024 Apr 11:73-81.

<https://doi.org/10.18311/jbc/2024/35481>

Another example can help to illustrate the fact of polymorphs strongly as follows:

BARSAGADE D, MASRAM P, Campus ME. POST EMBRYONIC CHANGES IN DIFFERENT CASTE OF WEAVER ANT, *OECOPHYLLA SMARAGDINA* (FABRICIUS) IN CENTRAL INDIA. Histopia Journal, 2017; 10 (1), 89-97.

The workers are described from the larval development stage, referred to as instars 1 to 5, to the pupal stage.

By comparison with our findings, we also found 5 types of diverse larvae corresponding closely to their respective pupae upon reaching individual shapes. These were not instar 1 to 5 but different individuals following a growth and development from egg to adult stage. Figs 5 E and 5 F represent the different individual in their larval and pupal shape – size, respectively. Each individual emerged as a distinct adult as shown in Fig 3 E and Figs 6 A-E. However, the cited articles failed to provide detailed descriptive photos showing the distinct polymorph up to the adult emergence final stage.

That was not a chronological life cycle history of changes from eggs, larvae, to pupae, of one group of major and minor workers only. It was the descriptive growth stage development of the distinctive life cycle history of the 5 identified polymorphs among workers.

To enhance the strength of this idea and fact, the following section was added to the research method:

2.2.6 Major - Intermediate workers evaluation – behaviors

As you can see, the intermediate workers (IM2, IM3) behave differently from the two identified big major worker classes, MBW and MIW.

Major big worker MBW and major intermediate worker are always in the first defensive line of territory boundaries or whenever there is an intrusion. They will face the intruder first, and the other intermediate workers 2 and 3 stay behind, defining layers of defense.

Recent publications give details on this aspect:

<https://www.cell.com/heliyon/fulltext/S2405-8440(24)02136-4#app-1> by Exélis et al., 2024. See section 2.3.3. Study 3: ground-dwelling foraging and territorial defense perimeter.

**Reviewer 2**

**Point 1. Missing Fig 14 A-B.**

That was a mistake in labelling as Fig 14 C-D. It is the same Figs A-B, corrected now.

**Point 2. In the Fig. 5 D, male drone larva should be indicated with black arrow not circle.**

Yes, it is indicated with a black arrow, thank you.

**Point 3. The Fig 11 D is broken.**

Yes, that's correct. Now it is adjusted accordingly. Thanks. New figures have been added since there was a mistake. The data were not standardized yet. It has been plotted again for the PCA.

**Point 4. Normal distribution – Test statistic**

**In line 406, please conduct a normality test and present the test statistic.**

The Shapiro-Wilk test for normality is applied, and it is found that the majority normally distributes body dimensions, while others are not. For simplicity at this stage, we assume that the data is normally distributed. If not, the Spearman's rank correlation coefficient can be used to calculate the correlation. However, the Box-Cox data transformation applied did not improve the data normality distribution. Hence, the data transformation was not necessary (See supporting information S3 and S4 Table).

**Normality Test: Shapiro-Wilk method is used to test for data normality.**

Shapiro Wilks Test: If p-value > 0.05, fail to reject H₀ → Data is normally distributed

| Ant Type | Dimension | p-value | Comment |
| --- | --- | --- | --- |
| MBW | Head width | 0.0995 | 1. 15 out of 25 ant worker dimensions are normally distributed. 2. 10 out of 25 ant worker dimensions are not normally distributed. 3. Since some variables are not normally distributed, ANOVA (parametric) is not preferred for comparing the means across different ant worker types. 4. Instead, the **Kruskal-Wallis test (non-parametric) is used to assess median equality**, as some variables are non-normally distributed, with p-values < 0.05. 5. Data homogeneity or equal variances must be checked for both normal and non-normal data using Levene's test. |
|  | Head length | 0.0178 |  |
|  | Thorax length | 0.0215 |  |
|  | Abdomen length | 0.0048 |  |
|  | Body length | 0.0663 |  |
| MIW | Head width | 0.7372 |  |
|  | Head length | 0.0924 |  |
|  | Thorax length | 0.8501 |  |
|  | Abdomen length | 0.2048 |  |
|  | Body length | 0.0814 |  |
| IW2 | Head width | 0.2376 |  |
|  | Head length | 0.0250 |  |
|  | Thorax length | 0.0142 |  |
|  | Abdomen length | 0.4445 |  |
|  | Body length | 0.2073 |  |
| IW3 | Head width | 0.0228 |  |
|  | Head length | 0.0073 |  |
|  | Thorax length | 0.6426 |  |
|  | Abdomen length | 0.0114 |  |
|  | Body length | 0.3680 |  |
| MW | Head width | 0.0448 |  |
|  | Head length | 0.2733 |  |
|  | Thorax length | 0.0866 |  |
|  | Abdomen length | 0.0371 |  |
|  | Body length | 0.5429 |  |

The values for asymmetry and kurtosis between -2 and + 2 are considered acceptable to prove normal univariate distribution (George & Mallery, 2010). Hair et al., (2010) and Bryne (2010) argue that data is considered to be normal if skewness is between – 2 and + 2, and kurtosis between -7 and + 7 (Supporting information S4 Table). Hence, the data can be considered normally distributed. Based on the descriptive statistics, it was found that the skewness is within -0.5 and 0.5. See section 3.0 statistical analysis of the manuscript with citations provided as supporting evidence.

| Variable | Mean | Median | Stdev | Skewness |
| --- | --- | --- | --- | --- |
| HW | 1.41 | 0.46 | 1.52 | -0.34 |
| HL | 2.21 | 0.57 | 2.26 | -0.48 |
| TL | 2.62 | 0.76 | 2.78 | -0.31 |
| AL | 2.66 | 1.09 | 2.51 | 0.42 |
| BL | 8.14 | 2.65 | 7.97 | 0.01 |

**Point 5.**

**In line 481, please present the test statistics. Eggs production (Queens fertility)**

Normality Test for ANOVA – Shapiro Wilk

> shapiro.test(dt$Nest)

Shapiro-Wilk normality test

data: dt$Nest

W = 0.92215, p-value = 0.109

The Tukey Honest Significant Difference (HSD) test is a post hoc multiple comparison test used to determine which group means are significantly different from each other after performing an ANOVA.

| Group | p-value | Sig./ Nsig. |  | Group | p-value | Sig./ Nsig. |  | Group | p-value | Sig./ Nsig. |
| --- | --- | --- | --- | --- | --- | --- | --- | --- | --- | --- |
| 2-1 | 1.0000 | Nsig. |  | **9-2** | **0.0091** | **Sig.** |  | 6-5 | 1.0000 | Nsig. |
| 3-1 | 0.1128 | Nsig. |  | **10-2** | **0.0007** | **Sig.** |  | **7-5** | **0.0044** | **Sig.** |
| 4-1 | 0.9870 | Nsig. |  | **4-3** | **0.0277** | **Sig.** |  | 8-5 | 0.1128 | Nsig. |
| 5-1 | 0.6854 | Nsig. |  | 5-3 | 0.8589 | Nsig. |  | 9-5 | 0.1447 | Nsig. |
| 6-1 | 0.7557 | Nsig. |  | 6-3 | 0.7996 | Nsig. |  | **10-5** | **0.0076** | **Sig.** |
| **7-1** | **0.0005** | **Sig.** |  | **7-3** | **0.0358** | **Sig.** |  | **7-6** | **0.0037** | **Sig.** |
| 8-1 | 0.8589 | Nsig. |  | **8-3** | **0.0131** | **Sig.** |  | 8-6 | 0.1360 | Nsig. |
| **9-1** | **0.0102** | **Sig.** |  | 9-3 | 0.7780 | Nsig. |  | 9-6 | 0.1201 | Nsig. |
| **10-1** | **0.0007** | **Sig.** |  | 10-3 | 0.0636 | Nsig. |  | **10-6** | **0.0063** | **Sig.** |
| 3-2 | 0.0994 | Nsig. |  | 5-4 | 0.2346 | Nsig. |  | **8-7** | **0.0001** | **Sig.** |
| 4-2 | 0.9931 | Nsig. |  | 6-4 | 0.2788 | Nsig. |  | 9-7 | 0.3666 | Nsig. |
| 5-2 | 0.6370 | Nsig. |  | **7-4** | **0.0002** | **Sig.** |  | 10-7 | 1.0000 | Nsig. |
| 6-2 | 0.7092 | Nsig. |  | 8-4 | 0.9999 | Nsig. |  | **9-8** | **0.0014** | **Sig.** |
| **7-2** | **0.0004** | **Sig.** |  | **9-4** | **0.0028** | **Sig.** |  | **10-8** | **0.0001** | **Sig.** |
| 8-2 | 0.8929 | Nsig. |  | **10-4** | **0.0002** | **Sig.** |  | 10-9 | 0.5644 | Nsig. |

*Sig.: Significance, Nsig.: Non Significance

**Point 6.**

**In line 565, please present the test statistics.**

Kruskal-Wallis rank sum test

| Dimension | p-value | Comment |
| --- | --- | --- |
| Head width | 2.2e-16 | If p-value < 0.05, we reject H₀, meaning at least one group is different. This implies that the numerical variable (e.g., head width) varies by group (e.g., ant type). If a variable changes based on group membership, it suggests that the group influences the variable, indicating a relationship (dependence).  From the Kruskal-Wallis test, all p-values < 0.05 which indicate that different ant worker types have significantly different median dimensions, suggesting they might be dependent on their sub-caste. |
| Head length | 2.2e-16 |  |
| Thorax length | 2.2e-16 |  |
| Abdomen length | 2.2e-16 |  |
| Body length | 2.2e-16 |  |
| R language codes:  > kruskal.test(HW ~ Type, data = dt)  Kruskal-Wallis rank sum test  data: HW by Type  Kruskal-Wallis chi-squared = 93.22, df = 4, p-value < 2.2e-16  > kruskal.test(HL ~ Type, data = dt)  Kruskal-Wallis rank sum test  data: HL by Type  Kruskal-Wallis chi-squared = 84.87, df = 4, p-value < 2.2e-16  > kruskal.test(TL ~ Type, data = dt)  Kruskal-Wallis rank sum test  data: TL by Type  Kruskal-Wallis chi-squared = 91.288, df = 4, p-value < 2.2e-16  > kruskal.test(AL ~ Type, data = dt)  Kruskal-Wallis rank sum test  data: AL by Type  Kruskal-Wallis chi-squared = 95.08, df = 4, p-value < 2.2e-16  > kruskal.test(BL ~ Type, data = dt)  Kruskal-Wallis rank sum test  data: BL by Type  Kruskal-Wallis chi-squared = 95.059, df = 4, p-value < 2.2e-16 | | |

**Point 7.**

**In line 588, If there is a strong correlation between variables AL and BL, couldn't we consider these two variables to have collinearity?**

In a regression model, the variance inflation factor (VIF) is used to detect multicollinearity. Fitting the linear regression model, BL is chosen as the response variable, and the other body dimensions are predictors. Multicollinearity was assessed using the Variance Inflation Factor (VIF) to detect potential redundancy among predictors (Supporting information S6 Table) (Sulaiman, 2015; Shrestha, 2020). The VIF values ranged between 5 and 10, indicating moderate multicollinearity in most models. However, this level of multicollinearity is considered reasonably acceptable up to a value reaching 40 (O’Brien, 2007).

O’brien, R.M., 2007. A caution regarding rules of thumb for variance inflation factors. *Quality & quantity*, *41*, pp.673-690.

| **Predictors** | **Significant Predictors**  p-value < 0.05  **(Regression Model)** | **Normality Test**  **for residual**  **Shapiro-Wilks**  p-value > 0.05  **(Regression Model)** | **VIF**  VIF < 5, no multicol  5 < VIF < 10, moderate multicol  VIF > 10, high multicol  VIF > 20, severe multicol |
| --- | --- | --- | --- |
| HW | HW  R2 = 0.93 | p-value = 0.3 > 0.05 | NA |
| HL | HL  R2 = 0.86 | p-value = 1e-04  < 0.05 | NA |
| TL | TL  R2 = 0.93 | p-value = 0.02  < 0.05 | NA |
| AL | AL  R2 = 0.96 | p-value = 0.003  < 0.05 | NA |
| HW, HL | HW, HL  R2 = 0.94 | p-value = 0.02  < 0.05 | HW HL  7.53 7.53 |
| HW, TL | HW, TL  R2 = 0.95 | p-value = 0.004  < 0.05 | HW TL  10.1 10.1 |
| HW, AL | HW, AL  R2 = 0.98 | p-value = 0.02  < 0.05 | HW AL  7.66 7.66 |
| HL, TL | HL, TL  R2 = 0.93 | p-value = 8e-05  < 0.05 | HL TL  8.63 8.63 |
| HL, AL | HL, AL  R2 = 0.97 | p-value = 0.003  < 0.05 | HL AL  5.66 5.66 |
| TL, AL | TL, AL  R2 = 0.97 | p-value = 0.8  > 0.05 | TL AL  8.65 8.65 |
| HW, HL, TL | HW, TL  R2 = 0.95 | p-value = 0.003  < 0.05 | HW HL TL  11.60 9.93 13.29 |
| HW, HL, AL | HW, AL  R2 = 0.98 | p-value = 0.01  < 0.05 | HW HL AL  11.17 8.26 8.40 |
| HW, TL, AL | HW, TL, AL  R2 = 0.98 | p-value = 0.2  > 0.05 | HW TL AL  11.7 13.2 10.0 |
| HL, TL, AL | TL, AL  R2 = 0.98 | p-value = 0.4 > 0.05 | HL TL AL  8.98 13.74 9.01 |
| HW, HL, TL, AL | HW, AL, TL  R2 = 0.98 | p-value = 0.2 > 0.05 | HW HL TL AL  13.00 9.99 15.99 10.10  HL has moderate multicol. |

**Point 8.**

**In line 593, please present the test statistics.**

This is the test carried out for Figs 11 A-D.

```
> head(dt_numeric)
    HW   HL   TL   AL    BL
1 2.12 3.15 3.90 5.00 12.75
2 2.10 3.15 3.90 4.90 12.69
3 2.07 3.10 3.90 4.90 12.65
4 2.05 3.00 3.85 4.85 12.50
5 2.05 3.00 3.77 4.70 12.35
6 2.02 3.00 3.75 4.65 12.25
> dt_scaled <- scale(dt_numeric)  # Standardize the data
> head(dt_scaled)
           HW       HL       TL       AL       BL
[1,] 1.537185 1.657712 1.682016 2.139589 1.742367
[2,] 1.493841 1.657712 1.682016 2.048005 1.719709
[3,] 1.428826 1.569882 1.682016 2.048005 1.704603
[4,] 1.385482 1.394221 1.616054 2.002213 1.647958
[5,] 1.385482 1.394221 1.510516 1.864837 1.591313
[6,] 1.320466 1.394221 1.484132 1.819045 1.553549
```

```
> pca_model <- prcomp(dt_scaled, center = TRUE, scale. = TRUE)
> pca_model
Standard deviations (1, .., p=5):
[1] 2.1851018 0.3232818 0.2449906 0.2170055 0.1170779

Rotation (n x k) = (5 x 5):
          PC1         PC2          PC3        PC4         PC5
HW -0.4475830 -0.04778289  0.799618786  0.2847604  0.27732211
HL -0.4408355 -0.74998452 -0.386017288  0.3042535 -0.04009632
TL -0.4489580 -0.10328923  0.007978341 -0.8771555  0.13528720
AL -0.4456783  0.56625734 -0.458998824  0.2291913  0.46638495
BL -0.4529251  0.32237243  0.029272459  0.0664143 -0.82805023
```

**Interpretation of rotation (Eigenvectors or Loadings)**

Consider the absolute value of eigenvalues and the positive or negative sign indicate the direction. High absolute values refer to the variable which contributes significantly to that PC.

PC1 is influenced by all four variables since the loadings (0.44-0.45) are similar, it suggests they contribute almost equally to the first principal component. It also suggests that they are highly correlated and influence the dataset in a similar way.

PC2 is strongly associated with HL (-0.75) which captures the variation mainly in the HL.

Hence, PC1 is related to all variables (HW,HL,TL,AL,BL), and PC2 is mainly influenced by HL.

```
> summary(pca_model)
Importance of components:
                          PC1    PC2    PC3     PC4     PC5
Standard deviation     2.1851 0.3233 0.2450 0.21701 0.11708
Proportion of Variance 0.9549 0.0209 0.0120 0.00942 0.00274
Cumulative Proportion  0.9549 0.9758 0.9878 0.99726 1.00000
```

Interpretation:

PC1 explains 95.5% of variance, meaning it captures the most information.

PC2 explains 2.1%, making a total of 97.6% variance in just two PCs.

PC3, PC4 and PC5 explain little variance (2.4%), meaning they are less important.

Since PC1 and PC2 explain most of the variance, we can use them to visualize the data in 2D instead of 4D.

fviz_eig(pca_model) # Scree plot

PC1 explains the most variance (~72.77%), meaning it captures most of the variation in the dataset.

PC2 explains around 23.03%, adding important but less dominant variation.

PC3 and PC4 contribute much less (~4.25% and ~0.44%), suggesting they can be ignored.

From the scree plot, PC1 explains the most variance (95.5%) which means that it captures most of the variation in the dataset.

PC2 explains around 2.1%, adding important but less dominant variation.

fviz_pca_biplot(pca_model, label = "var", habillage = dt$Type, addEllipses = TRUE)

|  | Interpretation:  A biplot is a combination of a score plot and a loading plot in PCA. It helps to understand how the observations (data points) and variables (features) relate in a reduced-dimensional space (typically PC1 vs. PC2).  X-axis represent PC1, First principal component (explains the most variance) and Y-axis represent PC2, Second principal component (explains the second most variance).  MW tends to cluster separately. MBW and MIW overlap slightly but still form distinguishable groups. Hence, PC1 and PC2 can differentiate all the ant sub-castes well. |
| --- | --- |

fviz_pca_var(pca_model, col.var = "contrib", gradient.cols = c("blue", "red"))

|  | Arrow length show the strength of the variable’s contribution to PCs.  Longer arrows show that the variable has a stronger influence.  Shorter arrows show that less contribution to that PC.  Angle between arrows.  Smaller angles (closer to 0°) show highly correlated variables.  Acute angles (closer to 45°) show medium correlated variables.  Angles near 90° show weak or no correlation.  Angles near 180° (opposite directions) show negative correlation.  Interpretation:  HW and TL are closely aligned which show that they are highly correlated.  HL and AL, HL and BL, HL and HW, HL and TL, form a 45-degree angle, it means they are moderately correlated.  The arrows are pointed to the left which indicate that all variables (HW,HL,TL,AL,BL) have negative loadings on Principal Component 1 (PC1).  This means that higher PC1 scores correspond to lower values of these original features. |
| --- | --- |

```
dt_pca_df <- data.frame(PC1 = pca_model$x[,1],
                        PC2 = pca_model$x[,2],
                        Species = dt$Type)  # Ensure correct column name

ggplot(dt_pca_df, aes(x = PC1, y = PC2, color = Species)) +
  geom_point(size = 3) +
  theme_minimal() +
  ggtitle("Iris Data PCA Projection")
```

Interpretation:

The plot shows that the ant sub-castes are clearly clustered and well defined.

**Point 9. In line 723, there is not referernce of Hasegwa (2013).**

Yes correct. It is adjusted now with a new citation as follows below:

Hasegawa E, Watanabe S, Murakami Y. Evolution of the optimal reproductive schedule in the ant *Camponotus* (*Colobopsis*) *nipponicus* (wheeler): a demographic approach. Ecological entomology. 2018 Feb;43(1):126-33.

And supported with new citation: Franck, 1987.

Frank SA. Variable sex ratio among colonies of ants. Behavioral Ecology and Sociobiology. 1987 Mar;20:195-201.
